# Efficacy of different gingival graft de-epithelialization methods: A parallel-group randomized clinical trial

**DOI:** 10.1007/s00784-025-06365-7

**Published:** 2025-05-07

**Authors:** Furkan Din, Meltem Özdemir Kabalak, Birtan Tolga Yılmaz, Emre Barış, Hanife Avcı, Feriha Çağlayan, H. Gencay Keceli

**Affiliations:** 1 Department of Periodontology, Faculty of Dentistry, Hacettepe University, Sihhiye, Ankara, 06230 Turkey; 2 Private Practice, Izmir, Turkey; 3 Department of Oral Medicine, Infection and Immunity, Harvard School of Dental Medicine, Boston, MA USA; 4 Department of Dental Biomaterials, Institute of Health Science, Dokuz Eylül University, Izmir, Turkey; 5 Department of Oral Pathology, Faculty of Dentistry, Gazi University, Ankara, Turkey; 6 Department of Biostatistics, Faculty of Medicine, Hacettepe University, Ankara, Turkey

**Keywords:** Connective tissue graft, De-epithelialization, Harvesting, Gingival recession, Root coverage

## Abstract

**Objectives:**

The clinical outcomes of either extra-oral (eo) or intra-oral (io) de-epithelialized connective tissue graft (DE-CTG) techniques to the recipient site have not been fully elucidated yet. There is an ongoing debate regarding the possible complications caused by incomplete elimination of the epithelial layer. The aim of this study is to compare clinical effectiveness and de-epithelialization efficacy of ioDE-CTG and eoDE-CTG in recession treatment.

**Materials and methods:**

Forty patients, with a single gingival recession, were treated with coronally advanced flap combined with eo- or ioDE-CTG. Data consisted of surgical chair-time, recipient site periodontal and patient-based variables. To evaluate the efficacy of de-epithelialization, histological analyses were also performed via graft imprints.

**Results:**

There was no significant inter-group difference in terms of treatment outcomes. Aesthetic results were also similar for both groups. While chair-time was significantly shorter in the ioDE group, less epithelial remnants were showed with eoDE technique.

**Conclusions:**

Similar clinical outcomes were obtained with eo- and ioDE-CTG. While ioDE is less effective in removing the epithelial layer, no long-term complications due to the epithelial residues were detected. Both techniques can be recommended in recession treatment.

**Clinical relevance:**

This study demonstrates that extraoral and intraoral de-epithelialized connective tissue grafts in root coverage procedures led to similar clinical results without any further events. The study protocol was registered at ClinicalTrials.gov (NCT05494294).

**Supplementary Information:**

The online version contains supplementary material available at 10.1007/s00784-025-06365-7.

## Introduction

De-epithelialized (DE) connective tissue graft (CTG) harvesting technique is recommended by many authors in the treatment of soft tissue defects around teeth and implant. It is preferred over trap-door and single incision techniques, which increase the morbidity by creating deeper wounds [1–3]. Moreover, the dense, collagen-rich layer of DE-CTG is less prone to shrinkage during the healing period, thereby enhancing clinical outcomes at the recipient site [4]. However, epithelial remnants on the DE-CTG primarily due to the incomplete de-epithelialization are still a concern. Although several studies have reported adverse events associated with epithelial remnants beneath the surgical flap - such as cyst formation or suboptimal aesthetic results - there is still a lack of evidence regarding the most effective method eliminating the epithelial layer [5–8]. To mitigate the epithelial remnant risk, employment of magnification tools to enhance visibility during surgery, and various epithelial removal techniques such as laser ablation or scraping with the sharp instruments have been proposed [9–12].

Original DE-CTG method, also defined as extraoral DE-CTG (eoDE-CTG), is consisted of harvesting a graft including both the epithelium and the connective tissue layer, then, de-epithelialization of the graft extra-orally. The eoDE method, when used with magnification, provides clinicians with a clear view to identify and remove epithelial remnants, while also determining the border between epithelium and connective tissue layers. Alternatively, intraoral DE-CTG (ioDE-CTG) involves a de-epithelialization process before harvesting, offers the advantages of easier manipulation [13, 14], reduced chair-time [10], ability to see uniform bleeding as a subjective indicator of an effective epithelium removal [9, 15], and shorter interruption of the blood supply of the graft.

Although studies focusing on the pain and root coverage outcomes of different de-epithelialization approaches are available [10, 16, 17], the literature, underscore the need for further research to explore the effect of epithelial remnants [12, 13, 18]. In particular, there are no studies comparing eoDE with ioDE in terms of both epithelial remnants and clinical outcomes together. Thus, the aim of the study was to comprehensively evaluate the treatment outcomes and de-epithelization efficacy of ioDE by comparing with eoDE technique in recession treatment under the hypothesis of ioDE technique would offer better clinical outcomes with less epithelial remnants on the CTG.

## Materials and methods

### Study design and patient population

The randomized, controlled, parallel-group, single-blinded study with 12-month follow-up was carried out by considering the CONSORT guidelines reporting parallel-group randomized trials [19]. The protocol was approved by the Ethical Committee of Hacettepe University, Ankara, Türkiye (KA-20039), and registered at ClinicalTrials.gov (NCT05494294). The study was conducted at Hacettepe University, Department of Periodontology, between July 2021 and July 2023. Patients requiring CTG for recession treatment were screened and who met the following inclusion criteria and provided written informed consent were included (M.Ö.K.).

#### Inclusion criteria

- age between 18 and 60,
- agreement to participate in the study after reading the informed consent,
- having vital incisor, canine, or premolar teeth with single, >2 mm-depth RT1 or RT2 recession [20],
- presence of an identifiable cemento-enamel junction (CEJ),
- completed non-surgical periodontal treatment,
- full-mouth plaque and bleeding scores < 15%,

#### Exclusion criteria

- uncontrolled systemic diseases, pregnancy or lactation,
- use of drugs causing gingival enlargement,
- presence of a donor site where the slide cannot be adapted for imprinting due to it is concavity or shallowness,
- uncontrolled endodontic problem in the involved tooth,
- previous periodontal plastic surgery in the relevant sites,
- root concavity, caries, non-carious lesions, restorations, mobility, and malocclusion in teeth undergoing treatment,
- smoking.

### Sample size calculation

G*Power software was used to calculate the sample size. By predicting the inter-group recession depth (RD) difference as 0.50 mm [21] and the pooled variance as 0.46 mm^2^, a large effect size was determined (cohen d = 1.086). For an effect size difference of 1.086 between the groups, 90% power and an alpha error of 5% were calculated with at least 19 participants in each group.

### Study groups, randomization, and allocation concealment

Patients who met the inclusion criteria, were informed verbally, and gave written informed consent were included. Randomization was performed in a 1:1 random allocation ratio using a computer-based randomization list by one of the authors (M.Ö.K.). Sealed opaque envelopes numbered sequentially and kept until the day of the surgery. After the administration of local anesthesia, a person did not involve the study communicated the assigned group to operator. ioDE group (*n* = 20) was treated with coronally advanced flap (CAF) combined with intraoral de-epithelialized CTG, while eoDE group (*n* = 20) was also treated with the CAF applied with CTG obtained by extraoral de-epithelialization.

### Treatment protocol

**Fig. 1:**
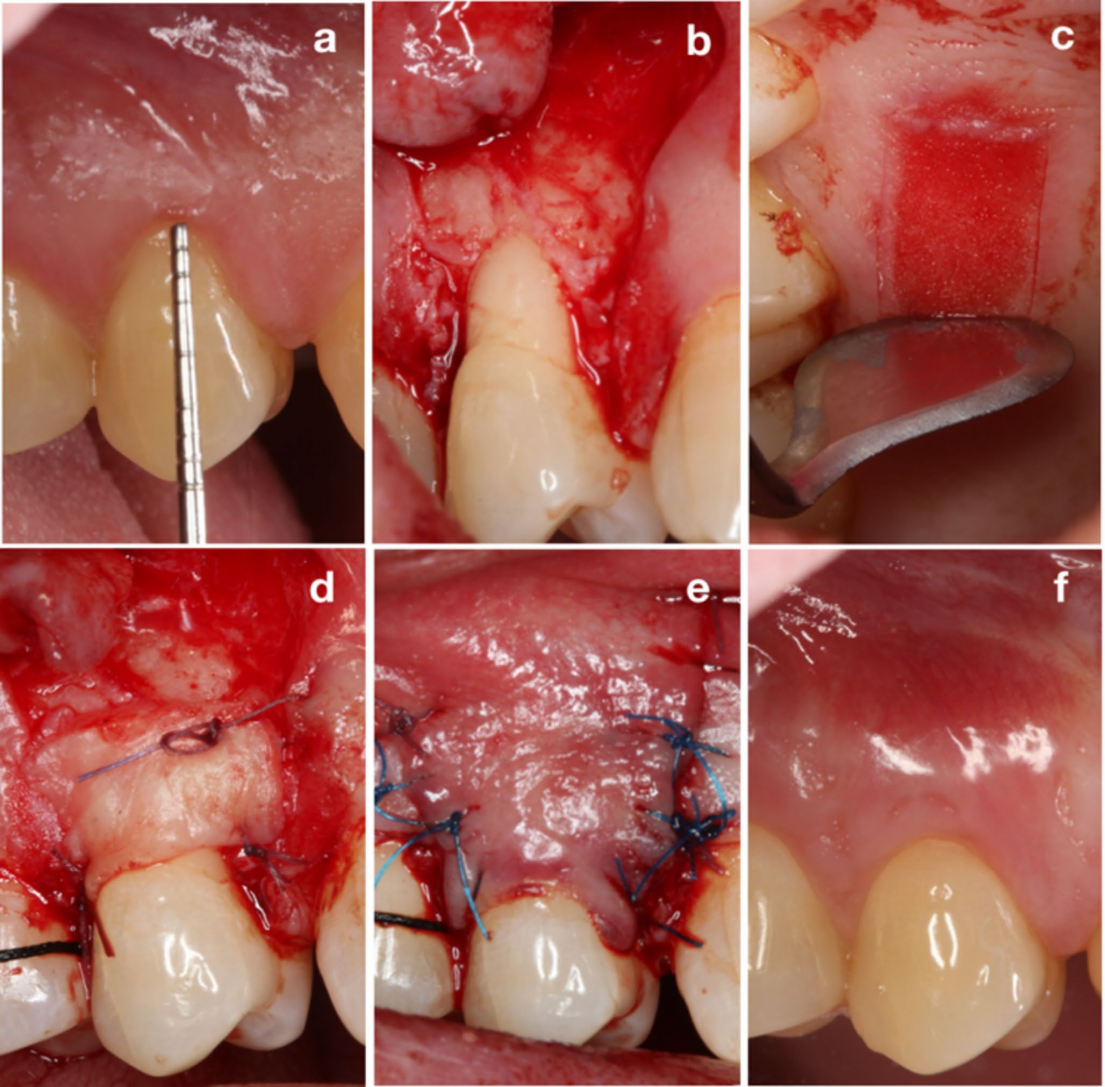
Treatment of gingival recession with intraoral de-epithelialized connective tissue graft (ioDE-CTG) in combination with coronally advanced flap (CAF) (**a**) baseline, (**b**) flap design, (**c**) intraoral de-epithelialization of the graft with Kirkland knife, (**d**) placement of the graft (**e**) suturation (**f**) 12 months after surgery

**Fig. 2:**
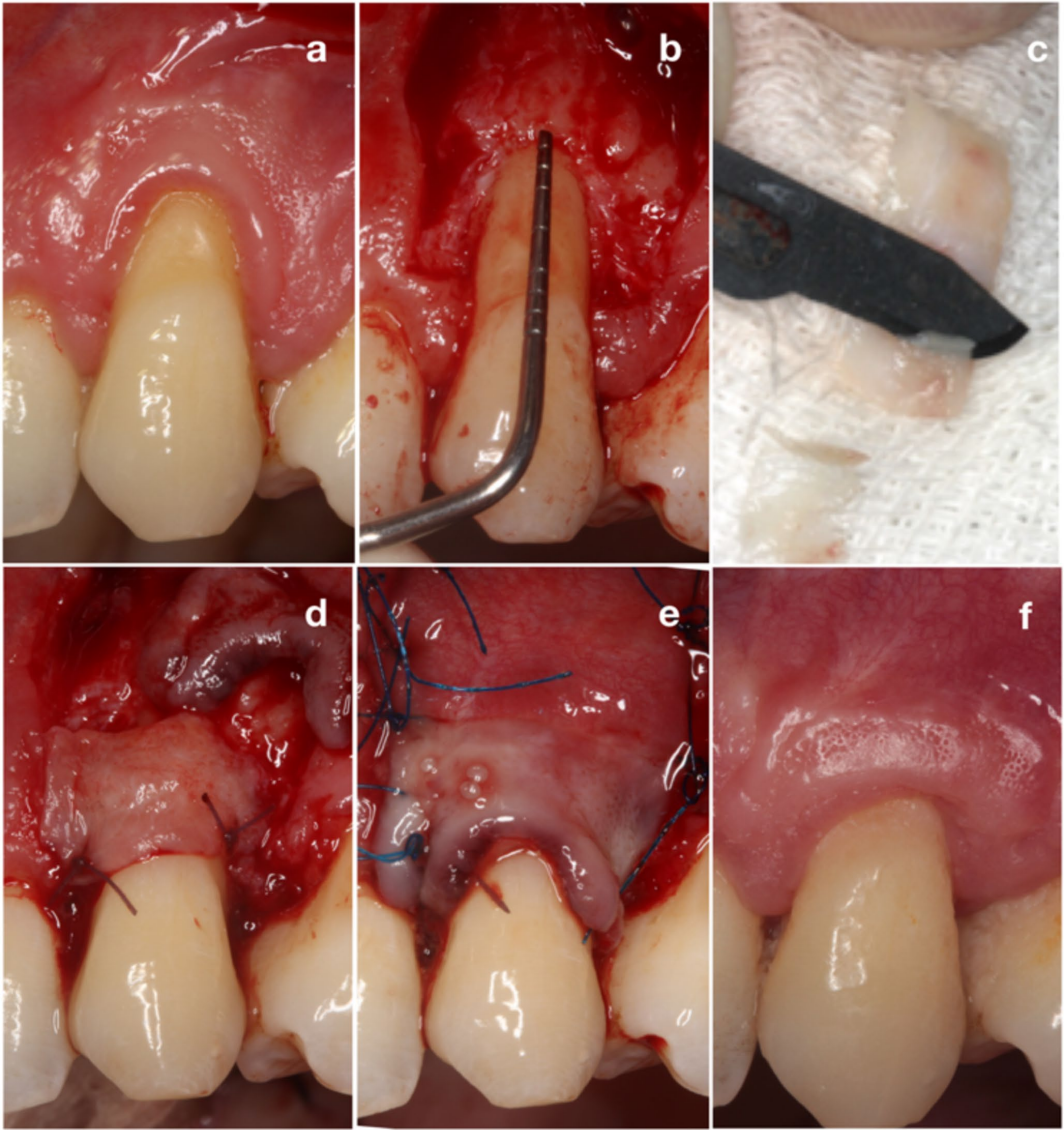
Treatment of gingival recession with extraoral de-epithelialized connective tissue graft (eoDE-CTG) in combination with coronally advanced flap (CAF) (**a**) baseline, (**b**) flap design, (**c**) extraoral de-epithelialization of the graft with a scalpel, (**d**) placement of the graft (**e**) suturing (**f**) 12 months after surgery

All interventions were performed by a periodontist (F.D.) with five years of experience in periodontal surgery. Prior to the surgery, exposed root surface in the recipient site was planed with curettes (5/6 Gracey Curette, Hu-Friedy, Chicago, IL) in both groups, no other root surface modification or biomaterial was applied throughout the surgery. Bilaminar (CTG + CAF) technique was used for treatment of recessions [22], and case examples were shown in Figs. 1 and 2. DE-CTG harvested to use for CAF procedure. Donor area was chosen within the lateral palate between the distal of the canine and the first molar. In the ioDE group, after determining the graft borders with a #15 C blade, intraoral de-epithelialization was performed with a scraping movement by using a Kirkland knife (15/16 Kirkland Periodontal Knife, Hu-Friedy, Chicago, IL) under 2.5x magnification (Fig. 1c). Scraping procedure continued until a depth of at least 1 mm compared to the adjacent tissue was reached and bleeding was observed throughout the entire graft area in order to be sure the fully elimination of the epithelium [9, 13]. Then, a split-thickness graft was obtained from the area by dissecting with a #15 blade kept parallel to the external palatal surface. In the eoDE group, after marking the graft borders with a #15 C blade epithelialized graft was harvested from the donor site using the #15 blade and de-epithelialized on a moistened sterile gauze under frequent irrigation of saline by using a 15 C blade as described [1] (Fig. 2c). In both groups, after bleeding control with moist gauze compress, the donor site wound was covered with gelatin sponge and the stabilization of the material achieved by cross-mattress sutures (5 − 0 Polypropylene, Propilen^®^ Dogsan Inc, Trabzon, Türkiye).

After trimming, the obtained graft was placed on the root surface and sutured to the anatomical papillae with synthetic, bioabsorbable, multifilament sutures (6 − 0 Polyglactin 910, Vicryl™ Ethicon USA, LLC, Somerville, USA) (Figs. 1 and 2d). The surgical flap was positioned with a sling suture closed by simple sutures (6 − 0 Polypropylene, Propilen^®^ Dogsan Inc, Trabzon, Türkiye) to cover the graft and the anatomical papilla regions completely (Figs. 1 and 2e.)

After surgery, each patient was given instructions for postoperative care for both donor and recipient sites. Tooth-brushing was discontinued around the surgical sites until the suture removal. 0.12% chlorhexidine digluconate (Kloroben, Drogsan, Türkiye) oral rinse was used twice a day for 14 days. Analgesics (Brufen, 400 mg) were prescribed for use in case of need. The sutures in the palate were removed 7 days after the surgery while they were kept in the recipient site for 14 days.

### Data collection

The data were collected by one calibrated author (H.G.K.) blinded to the treatment allocation. To assess intra-examiner reproducibility, RD measurements at another set of 20 recession defects were evaluated twice for each patient within a 48-hour interval. The kappa statistics was used to determine the agreement and showed a similarity of 92% at the millimeter level.

#### Demographics

Patients’ age, gender, included teeth, and type of recession information were recorded.

#### Graft dimensions

Thickness, width (mesial–distal dimension), and height (apical-coronal dimension) of the graft was measured just before placing the graft in the recipient site. While thickness was measured with a standardized caliper (Kohdent 7243, Kohdent Roland Kohler Medizintechnik, Stockach, Freiburg, Germany), a periodontal probe (UNC15, Hu-Friedy, Chicago, IL, USA) was used for measurements of width and height.

#### Periodontal variables (*BL*,* T3*,* T6*,* T12)*

The following clinical measurements were made using a periodontal probe (UNC15, Hu-Friedy, Chicago, IL, USA);

- Recession depth (RD), distance from CEJ to gingival margin (primary outcome measure).
- Recession width (RW), horizontal distance between mesial and distal gingival margins measured by keeping the probe tangent to CEJ.
- Clinical attachment level (CAL), distance from CEJ to the bottom of the pocket.
- Keratinized tissue width (KTW), distance from gingival margin to mucogingival junction.
- Gingival thickness (GT), under local anesthesia, distance from gingival surface to the underlying hard tissue was measured by inserting a 20 N endodontic spreader into the mid-buccal region 2 mm apical to the gingival margin and placing a stopper at the end point where it advanced. After the spreader was removed, the distance between the tip and the stopper was measured with a standardized caliper (Kohdent 7243, Kohdent Roland Kohler Medizintechnik, Stockach, Freiburg, Germany).
- Vestibular depth (VD), distance from CEJ to the deepest region of the vestibular sulcus was determined with the aid of a periodontal probe held horizontally.

Using these measurements, CAL gain, KTW change, and VD change from baseline to 12 months were calculated. RC% was determined by calculating the percentage of RD differences between baseline and 12 months relative to baseline RD. Complete root coverage was recorded by calculating the number and percentage of defects with 100% RC at 12 months.

#### Patient-based and other variables

While dentin hypersensitivity and postoperative discomfort that was reported by the patients, the remaining variables were scored and recorded by a clinician (H.G.K.) as follows:

- Surgical chair-time: was measured starting from the first incision to the last suture using a chronometer.
- Wound healing index *(2w)* 1: uneventful recovery, mild edema, erythema, discomfort; no flap dehiscence and suppuration, 2: uneventful recovery, edema, erythema, discomfort; no flap dehiscence and suppuration, 3: inadequate healing, significant edema, erythema, discomfort; flap dehiscence and suppuration present/absent in the recipient site [23].
- Aesthetic evaluation *(6* *M)* was performed by scoring the gingival margin (0, 3, or 6 points), as well as marginal tissue contour, soft tissue texture, mucogingival junction alignment, and gingival color (0 or 1 point each). The scores were summed, with a total of 10 points representing an ideal aesthetic condition [24].
- Dentin hypersensitivity *(BL*,* 6M)* patients were instructed to record their tooth sensitivity in the recession site at baseline and 6th month follow-up according to five point scoring scale; 0: severe, 1: significant, 2: moderate, 3: mild, 4: none [25].
- Post-operative discomfort *(1w*,* 2w*,* 3w*,* 4w)* was recorded by applying a sensitivity test with an air spray for 5 s to the donor site and asking the patient to score between 0 and 10 based on a visual analogue scale of 100. “0” represents no pain and “10” extreme pain [26].

### Histological analysis

The imprint cytology method [27] was adapted to determine the de-epithelization efficiency. The number of squamous cells (stratified squamous epithelium cells beneath the keratin layer) and the number of keratin lamellae on the samples were counted from the histological imprints obtained by using adhesive positive-charged disposable slides (VWR, #82027-788, Radnor, PA) before and after de-epithelialization. In both groups, after determining the borders of the graft on the donor area, a sample from the epithelialized surface was taken by contacting the slide to the donor site before de-epithelialization (Online Resource 1). The contact between the slide and the tissue was ensured by observing the stasis view on the tissue surface. After that, in both groups, upper surface of the de-epithelialized grafts was contacted with another slide just before placing to the recipient site. Those slides were collected and processed by a different masked researcher (E.B.). After drying, the imprinted slides were fixed and stained with hematoxylin-eosin dye. Then, the total number of keratin lamellae, representing the cells shed from the uppermost surface of the epithelium, and squamous cells, representing the cells from the deeper layers of the epithelium, per imprinted surface marked with permanent marker was counted in triplicate under x200 magnification using the image analysis program integrated into the light microscope (Leica QWin V3, Westlar-Germany) (20 samples before and 20 samples after de-epithelialization in each group). The success of the applied de-epithelialization method was assessed by determining the ratio of these cells before and after de-epithelialization.

### Statistical analysis

Normality of the numerical variables was examined using the Shapiro-Wilk test. Continuous variables are reported as mean ± standard deviation or median (25.percentile–75.percentile), as appropriate whereas mean ± standard deviation presentation was used as well as for the clinical measurements that did not show normal distribution to compare the outcomes with the relevant literature. Categorical variables were presented with frequency and percentage values. Qualitative variables were compared between eoDE and ioDE groups by using the chi-square test. Inter-group comparisons were made using either Student’s t or Mann–Whitney U test. Within-group comparisons of the continuous variables were performed using Wilcoxon or Friedman test, as appropriate. Correlations between RD, RW, KTW, vestibule depth, E-DE variables, and RC% were evaluated with Spearman coefficients. A two-tailed *p* < 0.05 was used for statistical significance (SPSS 26.0, SPSS INC., Chicago, Illinois, USA).

## Results

**Fig. 3:**
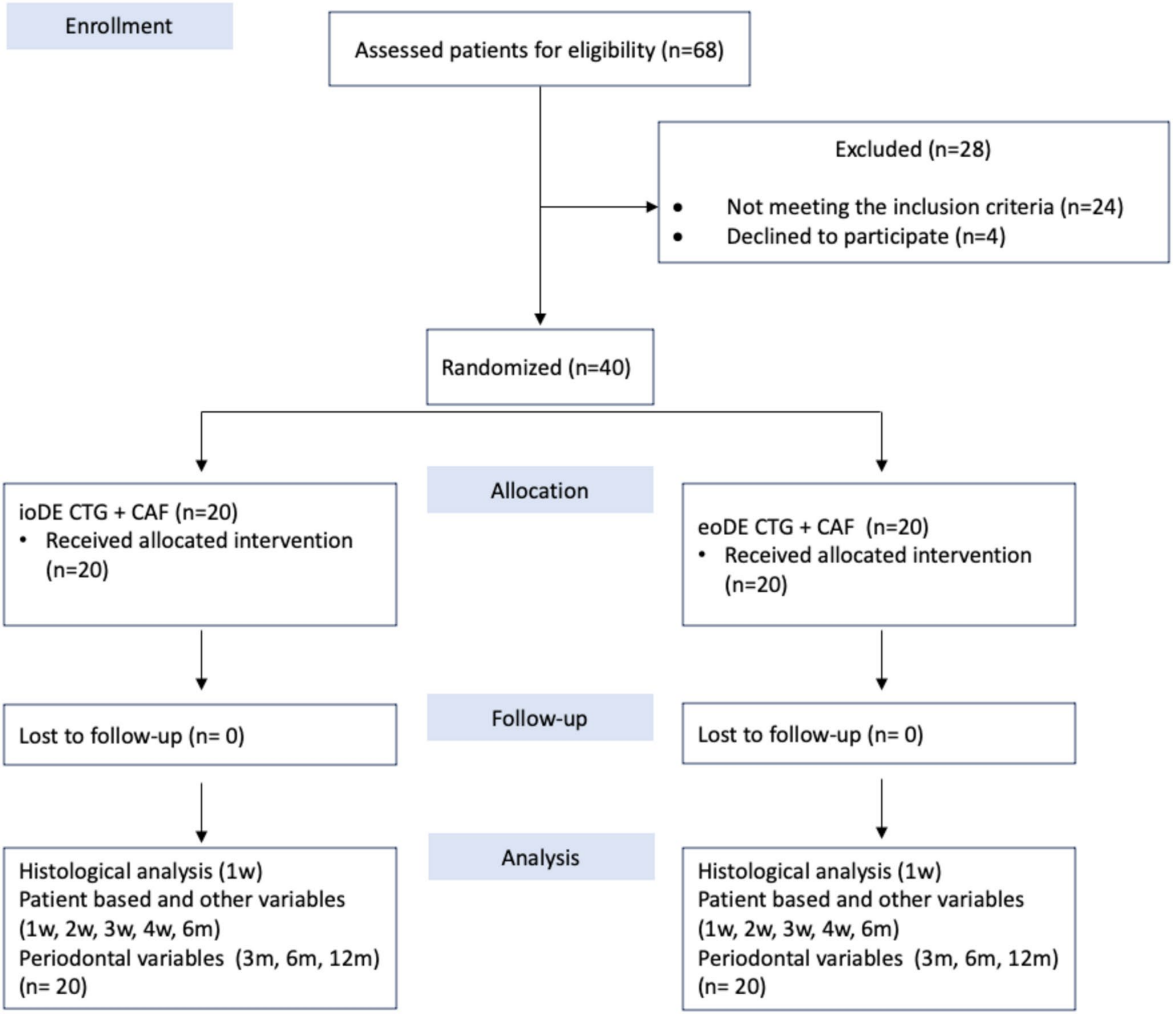
Flow chart of the study

In total, 68 referred patients were screened, 40 patients (*n* = 20 for each group) aged between 19 and 59 years who met the inclusion criteria were included and completed the follow-up with no drop-outs (Fig. 3). No long-term complications were seen in both groups. While the surgical chair-time was 34.76 ± 4.07 min in the ioDE group, it was higher in the eoDE group (38.19 ± 3.28 min) (*p* = 0.006).

### Demographic data, clinical characteristics, and graft dimensions

No statistically significant inter-group difference was detected for gender, age, recession type, recession site, and graft dimensions. (Table 1)

### Periodontal variables

Except vestibular depth, all periodontal variables showed significant improvement in both groups throughout the follow-up (*p* < 0.001), and no significant inter-group difference existed at baseline or follow-up. Mean RC% was 76.9 (eoDE) and 80.1 (ioDE) (*p* = 0.429). 35% (eoDE) and 65% (ioDE) of complete root coverage were obtained with no inter-group difference (*p* = 0.058). 1.03 mm (eoDE) and 0.98 mm (ioDE) mean GT increase was present (*p* > 0.05). While vestibular depth change was not significant in the eoDE group, a remarkable difference was present in the ioDE group from baseline to *12M* follow-up (*p* = 0.012) (Table 2).

**Table 1:** Demographic data, clinical characteristics, and graft dimensions

		eoDE	ioDE	Total	*p* value
		(*n* = 20)	(*n* = 20)	(*n* = 40)	
Age					
		44.0 ± 10.8	42.9 ± 10.1	43.4 ± 10.3	0.753^a^
Gender	Male (*n*, %)	8 (66.7%)	4 (33.3%)	12 (100%)	0.168^b^
	Female (*n*, %)	12 (42.9%)	16 (57.1%)	28 (100%)	
Recession type	RT-I (*n*, %)	12 (44.4%)	15 (55.6%)	27 (100%)	0.311^b^
	RT-II (*n*, %)	8 (61.5%)	5 (38.5%)	13 (100%)	
Recession site	Central Incisor (*n*, %)	7 (63.6%)	4 (36.4%)	11 (27.5%)	0.600^b^
	Lateral Incisor (*n*, %)	3 (75%)	1 (25%)	4 (10%)	
	Canine (*n*, %)	5 (41.7%)	7 (58.3%)	12 (30%)	
	First Premolar (*n*, %)	4 (36.4%)	7 (63.6%)	11 (27.5%)	
	Second Premolar (*n*, %)	1 (50%)	1 (50%)	2 (5%)	
Graft dimensions	Graft thickness	1.54 ± 0.2	1.53 ± 0.2	1.53 ± 0.2	0.856 ^a^
	Graft width	9 (8.0–10.0)	9.0 (8.0–9.5)	9.0 (8.0–9.5)	0.820^c^
	Graft height	6.0 (5.5–7.0)	6.0 (6.0–7.0)	6.0 (6.0–7.0)	0.512^c^

Data are presented as mean ± SD, median (25. percentile– 75. percentile), or frequency (percent).

^a^: Student t test

^b^: Chi-squared test

^c^: Mann-Whitney U test

eoDE, extraoral de-epithelialized connective tissue graft group; ioDE, intraoral de-epithelialized connective tissue graft group

**Table 2:** Recipient site variables

		eoDE (*n* = 20)	ioDE (*n* = 20)	*p* ^1^
RD (mm)	Baseline	3.0 (3.0–4.0) 3.4 ± 1.0	3.0 (2.3–3.5) 3.1 ± 1.0	0.414^a^
	3 months	1.0 (0.0-1.3) 0.8 ± 0.9	0.3 (0.0-1.5) 0.9 ± 1.3	0.779^a^
	6 months	1.0 (0.0-1.5) 0.9 ± 1.0	0.0 (0.0-1.8) 1.0 ± 1.4	0.820^a^
	12 months	1.0 (0.0–1.0) 0.8 ± 0.7	0.0 (0.0–1.0) 0.8 ± 1.3	0.289^a^
	p^2^	< 0.001^b^	< 0.001^b^	
RW (mm)	Baseline	3.0 (3.0–4.0) 3.4 ± 0.8	3.0 (3.0–4.0) 3.2 ± 0.9	0.512^a^
	3 months	2.0 (0.0–3.0) 1.8 ± 1.5	0.3 (0.0-2.5) 1.3 ± 1.5	0.341^a^
	6 months	2.0 (0.0–3.0) 1.6 ± 1.5	0.0 (0.0–3.0) 1.2 ± 1.4	0.478^a^
	12 months	2.0 (0.0–3.0) 1.7 ± 1.5	0.0 (0.0–3.0) 1 ± 1.4	0.174^a^
	p^2^	< 0.001^b^	< 0.001^b^	
CAL (mm)	Baseline	5.0 (4.0–5.0) 4.7 ± 1.1	4.0 (3.5–4.3) 4.1 ± 1.1	0.060^a^
	3 months	2.0 (1.0–3.0) 2.3 ± 1.3	1.8 (1.0–3.0) 2.2 ± 1.5	0.718^a^
	6 months	2.0 (1.0–4.0) 2.3 ± 1.4	2.0 (1.0-2.8) 2.2 ± 1.4	0.925^a^
	12 months	2.0 (1.0-2.8) 2.1 ± 1.3	1.0 (1.0-2.3) 2 ± 1.5	0.512^a^
	p^2^	< 0.001^b^	< 0.001^b^	
KTW (mm)	Baseline	3.0 (2.3–3.5) 3 ± 1.1	3.0 (2.5-4.0) 3.5 ± 1.4	0.369^a^
	3 months	6.0 (5.0-6.5) 5.6 ± 1.4	5.5 (3.8-6.0) 5.2 ± 1.8	0.461^a^
	6 months	5.8 (5.0–6.0) 5.4 ± 1.3	5.0 (4.0–6.0) 5.1 ± 1.7	0.529^a^
	12 months	5.8 (5.0–7.0) 5.5 ± 1.5	6.0 (4.0-6.5) 5.4 ± 1.9	0.718^a^
	p^2^	< 0.001^b^	< 0.001^b^	
GT (mm)	Baseline	1.0 (0.8-1.0) 0.9 ± 0.2	1.0 (1.0–1.0) 0.9 ± 0.2	0.799^a^
	3 months	2.0 (1.5-2.0) 1.8 ± 0.3	2.0 (1.5-2.0) 1.8 ± 0.4	0.968^a^
	6 months	2.0 (1.8-2.0) 1.9 ± 0.3	2.0 (1.5-2.0) 1.9 ± 0.5	0.640^a^
	12 months	2.0 (2.0–2.0) 1.9 ± 0.3	2.0 (1.5-2.0) 1.9 ± 0.4	0.925^a^
	p^2^	< 0.001^b^	< 0.001	
VD (mm)	Baseline	10.0 (8.0–11.0) 10.1 ± 3.5	10.0 (10.0–14.0) 11.1 ± 2.8	0.445^a^
	3 months	10.0 (8.0–12.0) 10 ± 2.9	10.0 (9.0–12.0) 10.3 ± 2.2	0.478^a^
	6 months	9.0 (8.0–11.0) 10 ± 2.7	10.0 (9.0–12.0) 10.4 ± 2.1	0.369^a^
	12 months	10.0 (8.0–12.0) 10 ± 3	11.0 (9.0–13.0) 10.7 ± 2.4	0.231^a^
	p^2^	0.612^b^	0.012^b^	
CRC%	Yes	7.0 (35.0)	13.0 (65.0)	
	No	13.0 (65.0)	7.0 (35.0)	0.058^c^
RC%	Baseline– 12 months	75.0 (100.0-66.7) 76.92 ± 24.58	100.0 (100 − 58.3) 80.13 ± 29.82	0.429^a^
CAG (mm)	Baseline– 12 months	3.0 (1.5-3.0) 2.57 ± 1.09	2.0 (1.5-3.0) 2.20 ± 0.965	0.231^a^
KTC (mm)	Baseline– 12 months	3.0 (2.0-3.8) 2.50 ± 1.37	2.0 (1.0–3.0) 1.875 ± 1.842	0.231^a^
VDC (mm)	Baseline-12 months	0.0 (-1.0-1.0) -0.10 ± 1.518	-1.0 (-1.0-0.0) -0.350 ± 1.899	0.289^a^

Data are presented median (25.percentile– 75.percentile) and mean ± SD.

p^1^: inter-group comparisons

p^2^: within-group comparisons (baseline − 12 months)

^a^: Mann-Whitney U test

^b^: Friedman test

^c^: Chi-squared test

eoDE, extraoral de-epithelialized connective tissue graft group; ioDE, intraoral de-epithelialized connective tissue graft group; RD, recession depth; RW, recession width; CAL, clinical attachment level; KTW, keratinized tissue width; GT, gingival thickness; VD, vestibular depth, RC, root coverage; CAG, clinical attachment gain; KTC, keratinized tissue width change; VDC, vestibular depth change

### Histological analysis

**Fig. 4:**
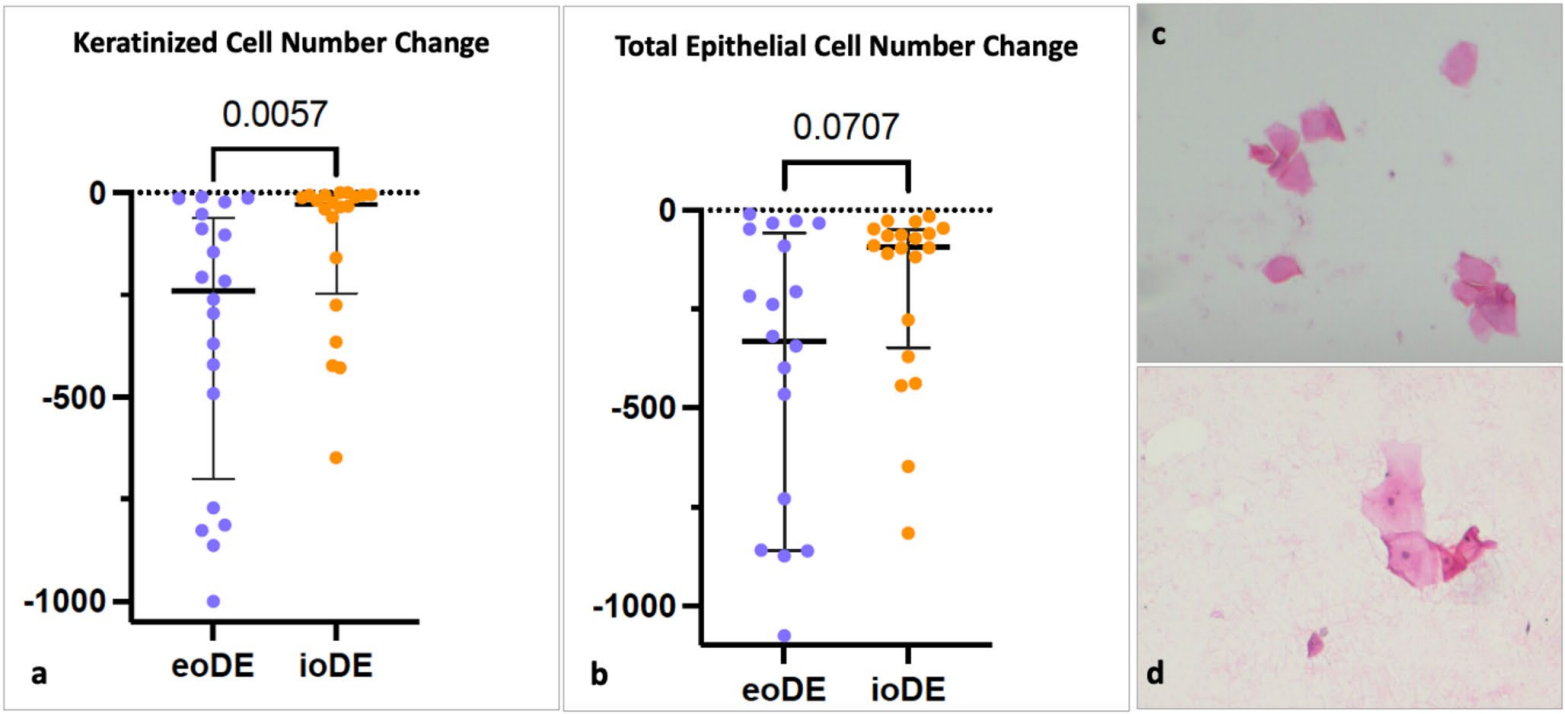
Histological analysis (**a**) number of keratin lamellae difference between before and after de-epithelialization (Mann–Whitney U test), (**b**) number of total epithelial cell difference between before and after de-epithelialization (Mann–Whitney U test), (**c**) keratin lamellae, (**d**) squamous cells

No epithelial remnants were observed in 3 out of the 20 samples after de-epithelialization in each group. The decrease in the number of keratin lamellae obtained by eoDE was higher than ioDE (eoDE-348.90 ± 331.56, ioDE-127.70 ± 191.78) (*p* = 0.006) (Fig. 4) with no inter-group difference in the ratio of keratin lamellae cell counts. Although fewer squamous cells were seen in the eoDE group, no statistical difference was found with the ioDE group in terms of squamous cell number and ratio before and after de-epithelialization.

### Correlations

When the effect of initial periodontal variables on RC% was examined, only vestibular depth showed a positive weak correlation (*p* = 0.026, r_s_=0.353) and no significant correlation existed between the difference in keratin lamellae number and RC% (Table 3).

**Table 3:** Correlations

	RD	RW	CAL	KTW	VD	E-DE(k)
RC%						
r_s_ p	-0.255 0.112	0.022 0.893	-0.103 0.525	0.176 0.278	0.353 **0.026**	0.246 0.155

r_s_: Spearman correlation coefficient

RC, root coverage; RD, recession depth; RW, recession width; CAL, clinical attachment level; KTW, keratinized tissue width; VD, vestibular depth; E-DE(k), keratin lamellae count difference pre/post de-epithelialization

### Patient-based and other variables

Wound healing index and aesthetic scores were similar for both groups (Table 4). While dentin hypersensitivity was higher in the ioDE group at baseline, the difference between the two groups disappeared *6M* after the surgery. Hypersensitivity decreased in both groups until *6M* follow-up (*p* < 0.001) with no inter-group differences (*p* = 0.185) (Table 4). Although comparison of the decrease did not show inter-group difference, post-operative discomfort disappeared earlier in the ioDE group (Table 5).

**Table 4:** Patient-based and other variables

	eoDE	ioDE		*p*
Wound healing index (2 weeks)	1 (1–2)	1 (1–3)		0.192^a^
Aesthetic evaluation (6 months)	8 (6–9)	8 (6–9)		0.459^a^
Dentin hypersensitivity (baseline)	3 (1–4)	2 (1–4)		**0.007** ^**a**^
Dentin hypersensitivity (6 months)	4 (1–4)	4 (2–4)		0.185^a^
p (within-group)	**< 0.001** ^**b**^	**< 0.001** ^**b**^		
Analgesic use (mg)	2181 (0-6000)	2400 (0-8000)		0.731^b^
Surgical chair-time (min)	38.19 ± 3.28	34.76 ± 4.07		0.006^c^

Data are presented median (25.percentile– 75.percentile) and mean ± SD

^a^: Mann-Whitney U test

^b^: Wilcoxon test

^c^: Student’s *t t*est

eoDE, extraoral de-epithelialized connective tissue graft group; ioDE, intraoral de-epithelialized connective tissue graft group

**Table 5:** Results of two way analysis of variance in repeated measures

	eoDE	ioDE	Time (F, *p*-value, $\:{\eta\:}^{2}$)	Group	Time $\:\times\:$ Group	
Post-op discomfort (1 week)	0.68 ± 1.49	0.50 ± 0.89				
Post-op discomfort (2 week)	0.11 ± 0.46	0.00 ± 0.00				
Post-op discomfort (3 week)	0.00 ± 0.00	0.00 ± 0.00				
Post-op discomfort (4 week)	0.00 ± 0.00	0.00 ± 0.00	6.172, < 0.001, 0.169	0.542, 0.506, 0.014	0.162, 0.922, 0.005	

eoDE, extraoral de-epithelialized connective tissue graft group; ioDE, intraoral de-epithelialized connective tissue graft group

## Discussion

This study aimed to compare eoDE or ioDE CTGs in recession treatment and revealed similar clinical and patient-based outcomes with histological results in favor of eoDE that did not support the study hypothesis. Comparable RC% were previously reported by Bakhishov et al. [17] (eoDE-91.7%) and Ozcelik et al. [10] (eoDE-95.9% vs. ioDE-96.3%) with the present study in which 76.9% and 80.1% mean RC% was obtained with eoDE and ioDE techniques. Besides the inter-group similarity that was compatible with those studies, a relatively less RC% in the present study might be associated with the inclusion of RT2 recessions as well as RT1 defects.

The present study showed significant KTW and GT increase after utilization of both techniques. Median keratinized tissue changes were 3 mm and 2 mm in the eoDE and ioDE groups, respectively, with no significant difference. In this respect, the outcomes were compatible with Ozcelik et al. [10]. Moreover, eoDE and ioDE techniques revealed 1.03 mm and 0.98 mm mean GT increase respectively, with no statistical difference similar to the same study [10]. From the plenty number of CAF-CTG studies focusing on vestibular depth as a critical variable for long-term success [28–30], the 1.08 mm mean vestibular depth increase obtained by Parlak et al. [30] was similar to the ioDE group of the present study which was higher than eoDE showing that the use of ioDE-CTG with CAF seems more advantageous than eoDE-CTG in terms of vestibular depth increase, possibly due to less shrinkage of the graft via shorter interruption of blood supply [4]. Moreover, only baseline vestibular depth, but not RD, RW, KTW, and CAL, was positively correlated with RC. This result was consistent with a recent study [29] supporting the influence and importance of vestibular depth on treatment results that should be confirmed with further controlled clinical and histological studies involving various surgical methods.

A reduced surgical chair-time promises a more economical, comfortable and less complicated process for the patient and the clinician. Here, mean surgical chair-time of ioDE (34.76 min) was slightly less than ioDE group of a previous study that used diode laser (36.72 min) [10], while closer surgical chair-time obtained in eoDE groups (38.19 and 39.19 min). Although ioDE with laser has shortened the surgical chair-time [31, 32], the slightly shorter duration obtained with ioDE with Kirkland in the present study may be explained with the operator-related differences as well as the optimal design of Kirkland knife which saved time in the ioDE process. Moreover, performing the de-epithelialization on an intact tissue is relatively practical and more time-efficient compared to eoDE made on an harvested piece of tissue that needs higher magnification and more delicate working that naturally slows the procedure [9, 13]. The present findings of reduced surgical chair-time with ioDE compared to eoDE supported this phenomenon. The earlier disappearance of the post-operative discomfort in the ioDE group (second week) than eoDE group (third week) also uncovered the time-related superiority of ioDE possibly associated with the later suturation of the donor site in the eoDE group that was applied shortly after graft de-epithelialization. Further studies including various de-epithelialization techniques and the time detection data is still necessary for consensus.

To date, a wide variety of tools and methods such as lasers, burs, back-action chisels, and bone scrapers have been used for ioDE [9, 10, 16–18, 33]. From those, ioDE with burs and lasers needs additional equipment and setting or have higher thermal damage risk [13, 34, 35] whereas bone scraper has incompatible design for the palatal donor site despite its high de-epithelialization speed [18]. As a previously untested alternative, the Kirkland periodontal knife is an easy-to-use, low-cost and time-efficient instrument for ioDE showing compatibility with the anatomical structure and morphology of the lateral palate. With Kirkland knife, it is also possible to obtain a graft with a smoother surface and to preserve the connective tissue layer to the maximum extent. In the present study, de-epithelialization with Kirkland knife shortened the de-epithelialization time, provided de-epithelialization at a level that did not cause clinical complications, and similar clinical results. However, for effectiveness during de-epithelialization, the Kirkland knife must be very sharp, therefore the need for sharpening must be evaluated. The present findings may also support its use in ioDE-CTG harvesting but should be confirmed with further trials comparing with other ioDE methods.

In the present study, keratin lamellae, representing the uppermost layer of the stratified squamous epithelium, and squamous cells, representing the deeper layers, were evaluated separately to assess whether the epithelium had been completely or partially removed. ioDE seemed less effective to remove epithelium based on the lower difference of keratin lamellae count before and after de-epithelialization (eoDE-348.90 ± 331.56 vs. ioDE-127.70 ± 191.78). This finding may be associated with the lack of visual discrimination of epithelial and connective tissue layers in the ioDE approach where it was more possible by detecting the color differences of these layers by using a magnification system in the eoDE group. Couso-Queiruga et al., [13] histologically compared eoDE and ioDE and found similar percentages of samples including epithelial remnants (ioDE-13.6%, eoDE-16.7%) which was notably less in the ioDE group (20%) of another study [12] compared with their eoDE group (40%). Azar et al. [4] found epithelial remnants in all eoDE samples with a median fraction of 6.01% to the total area. On the contrary, here, almost 85% of the samples had epithelial remnant remaining. The dissimilarity with the relevant literature may be attributed to the evaluation and analysis methods of different techniques to detect epithelial remnants [36–38]. Different from the cited literature we adopted the imprint cytology approach that allowed quantitative evaluation of the epithelial cells that may have increased the sensitivity of the evaluation. Clinically, the difference in keratin lamellae count did not correlate with RC showing the de-epithelialization efficiency of both methods did not influence the clinical outcomes as the general trend in the relevant literature [17, 33]. Although it is expected that the number of remaining keratin lamellae being higher in the ioDE group may disrupt graft integration and thus, affect GT, KTW and esthetic evaluation, no difference was observed between the groups in this study. Furthermore, no long-term complications due to epithelial residues were observed.

Presence of bleeding upon de-epithelialization is mostly preferred as an indicator of sufficient ioDE. Although, this visual finding may show the complete removal of epithelial layer deprived of vessels, profuse bleeding that spread over the remaining wound surface carrying possible epithelial remnants may mislead to complete elimination of the epithelial tissue [13]. Additionally, reduced visibility may also make harvesting the CTG more challenging in the ioDE method. Previous findings indicate that the epithelial remnants mostly reside on the superficial aspect of the graft after ioDE while there is a difficulty of removing the epithelium from the corners and boundaries of the graft in the eoDE technique [13]. To overcome these issues, in the ioDE technique, regardless of the presence of bleeding, at least 1 mm scraping in depth (involving the epithelium layer) was carried out by measuring with a probe based on the reported mean epithelial thickness of the lateral palate (364 μm, range from 111 to 619 μm) [39] where more attention was paid during de-epithelialization of the periphery of the graft in eoDE procedure. However, in this study, the presence of small amounts of squamous cells in both groups showed that complete de-epithelialization was not achieved.

Considering the post-operative patient comfort and local ethical reasons, harvesting a larger CTG to perform a detailed histological evaluation was not preferred and can be assumed as a limitation. The absence of an analysis regarding to local phenotypic (e.g., total palatal tissue thickness) or histomorphometric characteristics (proportion of lamina propria and submucosa) that were assessed in previous studies [13, 17] might also be considered as a shortcoming of the study. Instead, the de-epithelialization efficacy was evaluated by using the cytological imprint technique for the first time has provided clinicians with a new perspective as an easily applicable alternative method.

According to the present findings, both eoDE- and ioDE-CTG revealed similar clinical outcomes. eoDE leaves less epithelial residues than ioDE with no difference in clinical complications. Within the given limitations, it can be concluded that both techniques can be recommended in recession treatment. To determine the most reliable and most appropriate technique, more comprehensive histological evaluations should be conducted.

## Electronic supplementary material

Below is the link to the electronic supplementary material.

Supplementary Material 1

## Data Availability

No datasets were generated or analysed during the current study.
