# Supplementary figures and images for: Efficacy of different gingival graft de-epithelialization methods: A parallel-group randomized clinical trial
Source: Clin Oral Investig. 2025 May 7;29(6):289. doi: 10.1007/s00784-025-06365-7 (PMC12058915; doi:10.1007/s00784-025-06365-7)

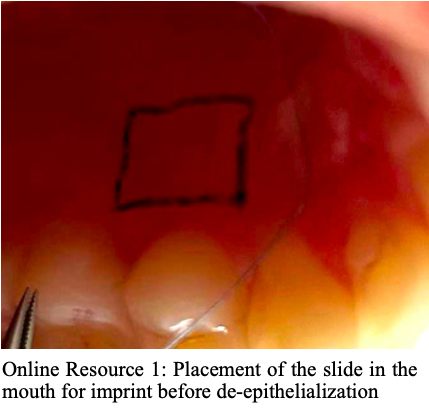

Supplement: Supplementary file 1 — Supplementary Material 1 [file 784_2025_6365_MOESM1_ESM.png]
